# Supplementary figures and images for: Surgical Treatment for Colorectal Cancer Partially Restores Gut Microbiome and Metabolome Traits
Source: mSystems. 2022 Mar 21;7(2):e00018-22. doi: 10.1128/msystems.00018-22 (PMC9040882; doi:10.1128/msystems.00018-22)

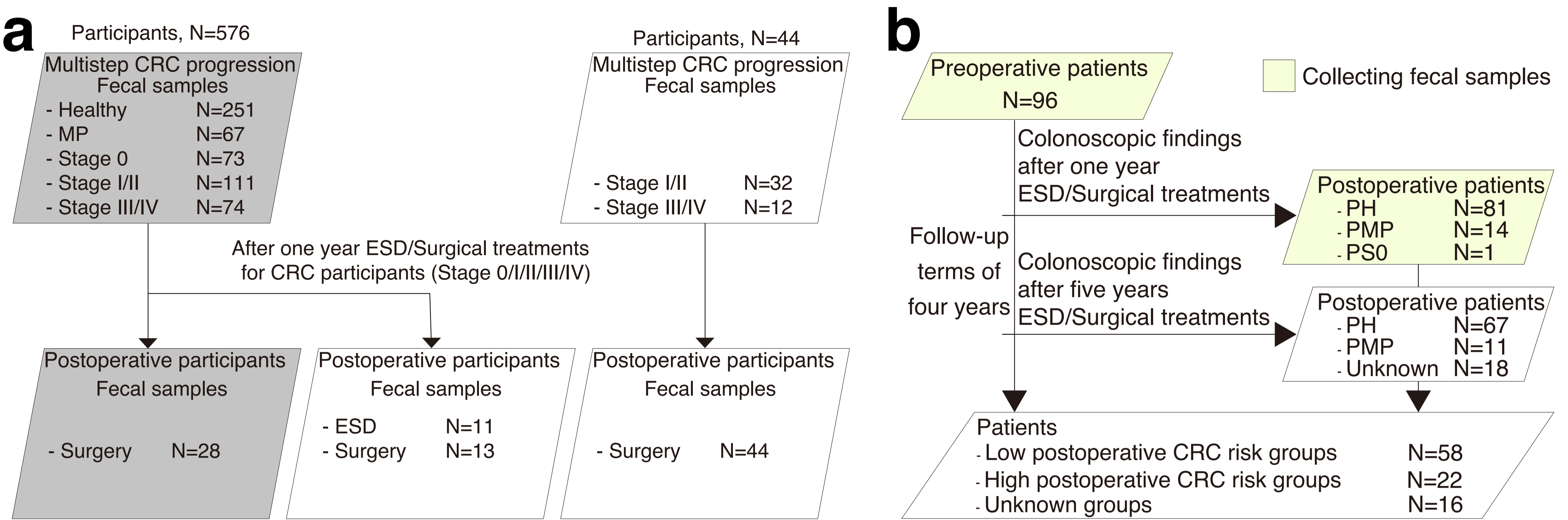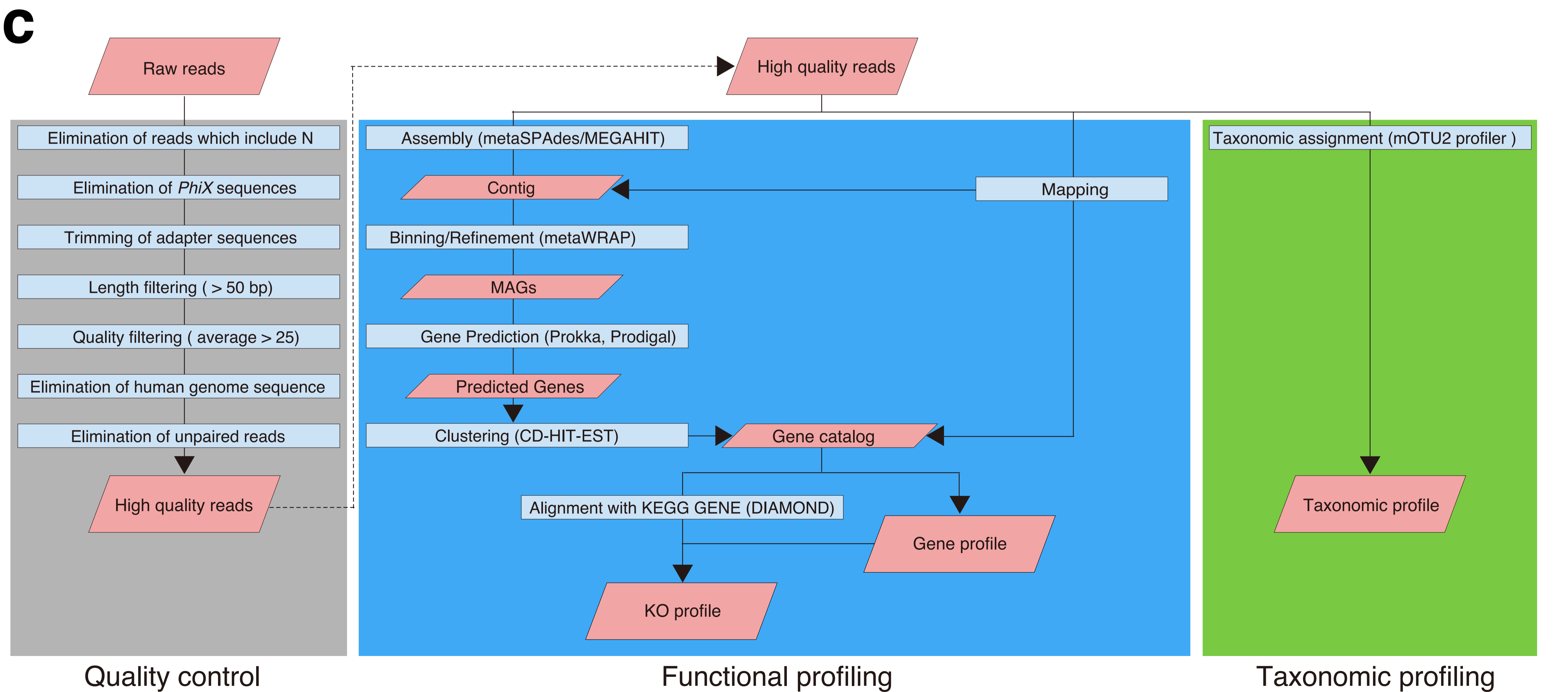

Supplement: FIG S1 [file msystems.00018-22-sf001.pdf]

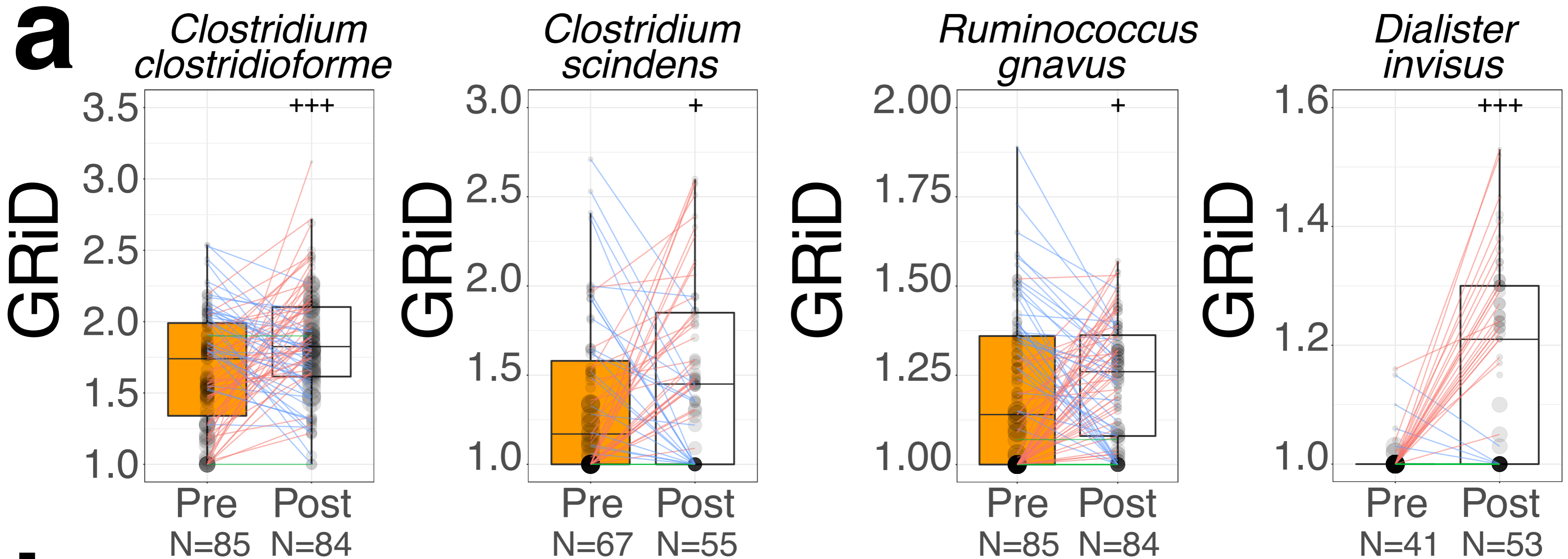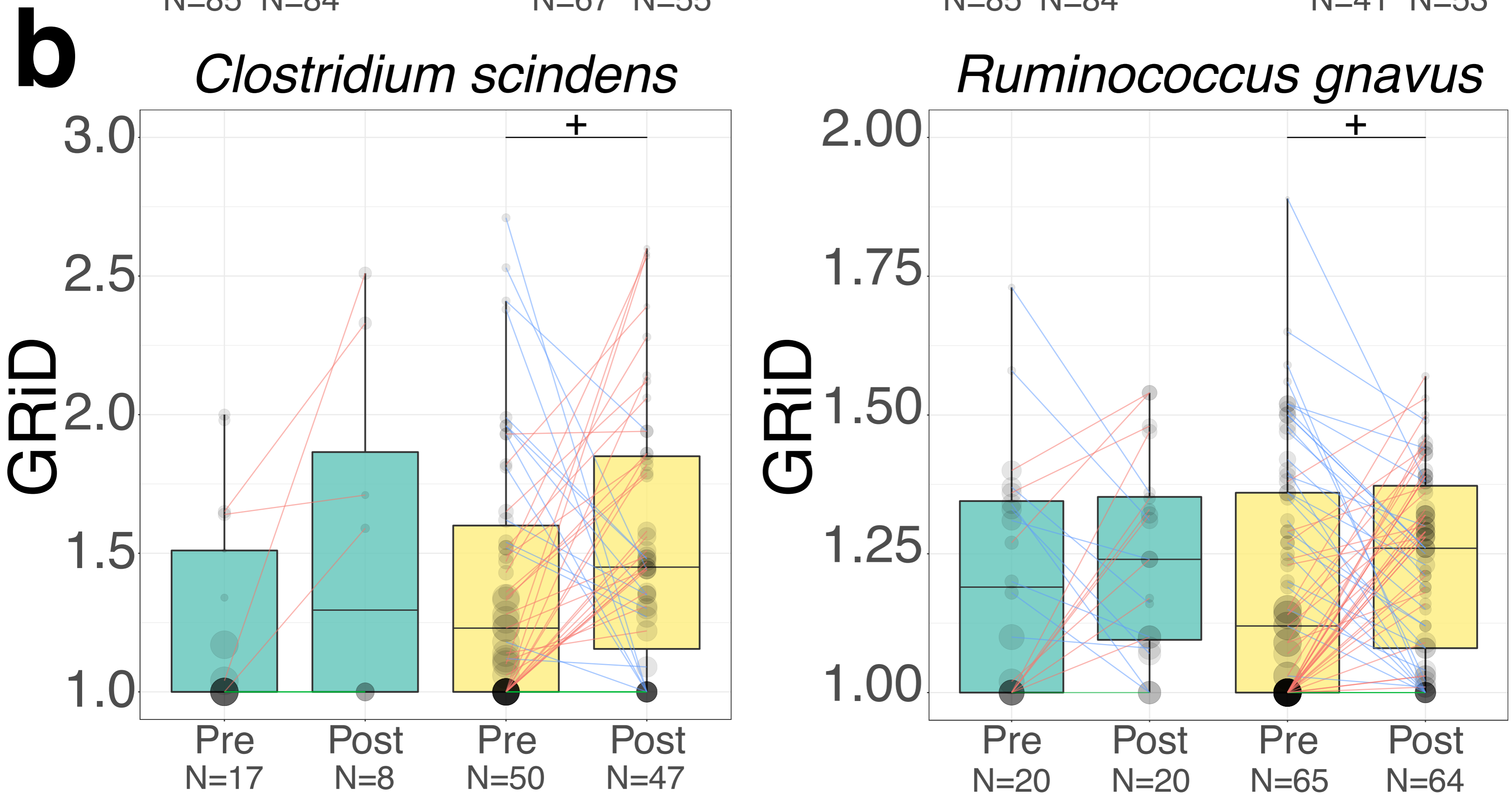

Supplement: FIG S2 [file msystems.00018-22-sf002.pdf]

**a**

Concentration ratio  
(DCA/Cholate)

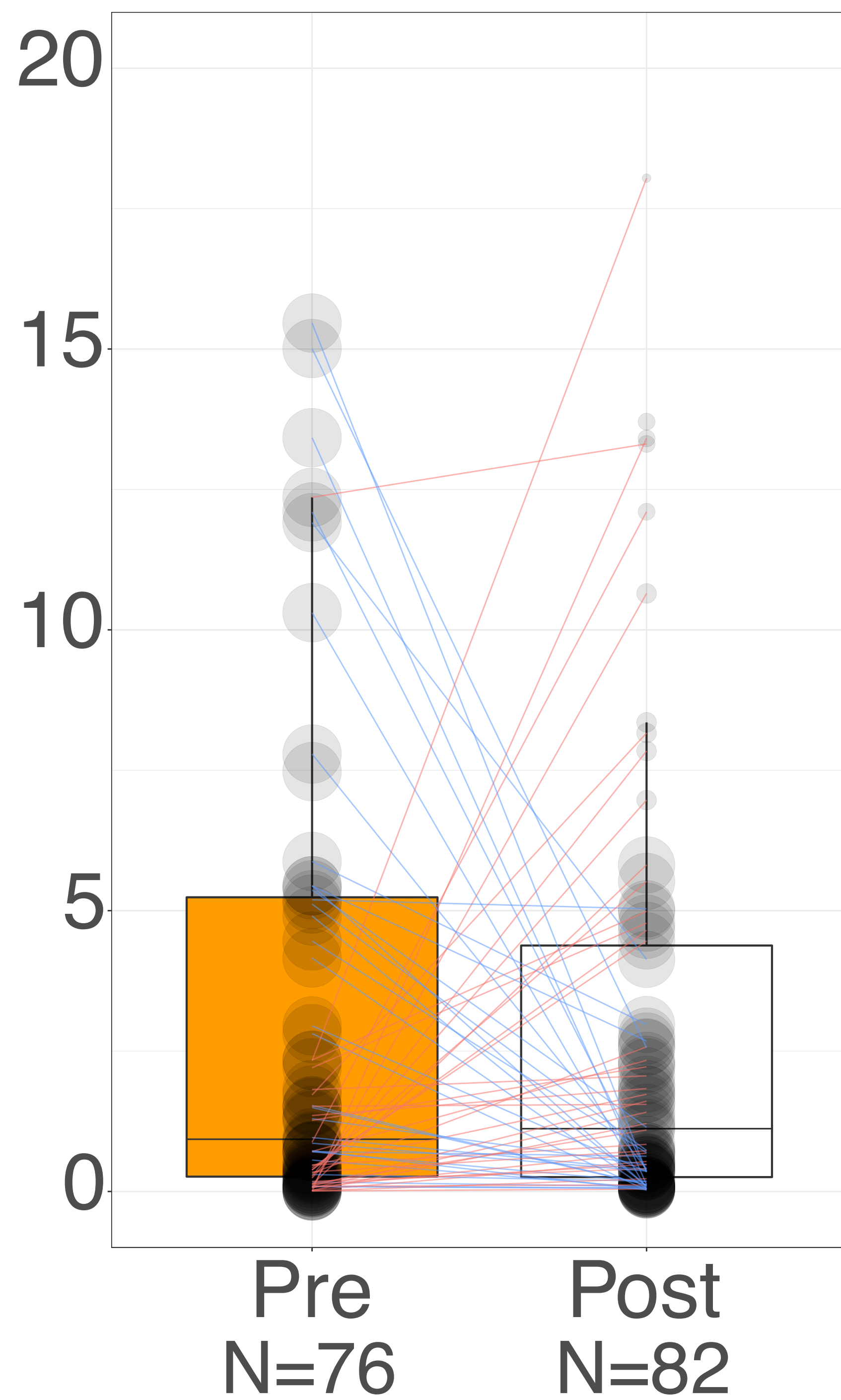

**b**

Concentration ratio  
(DCA/Cholate)

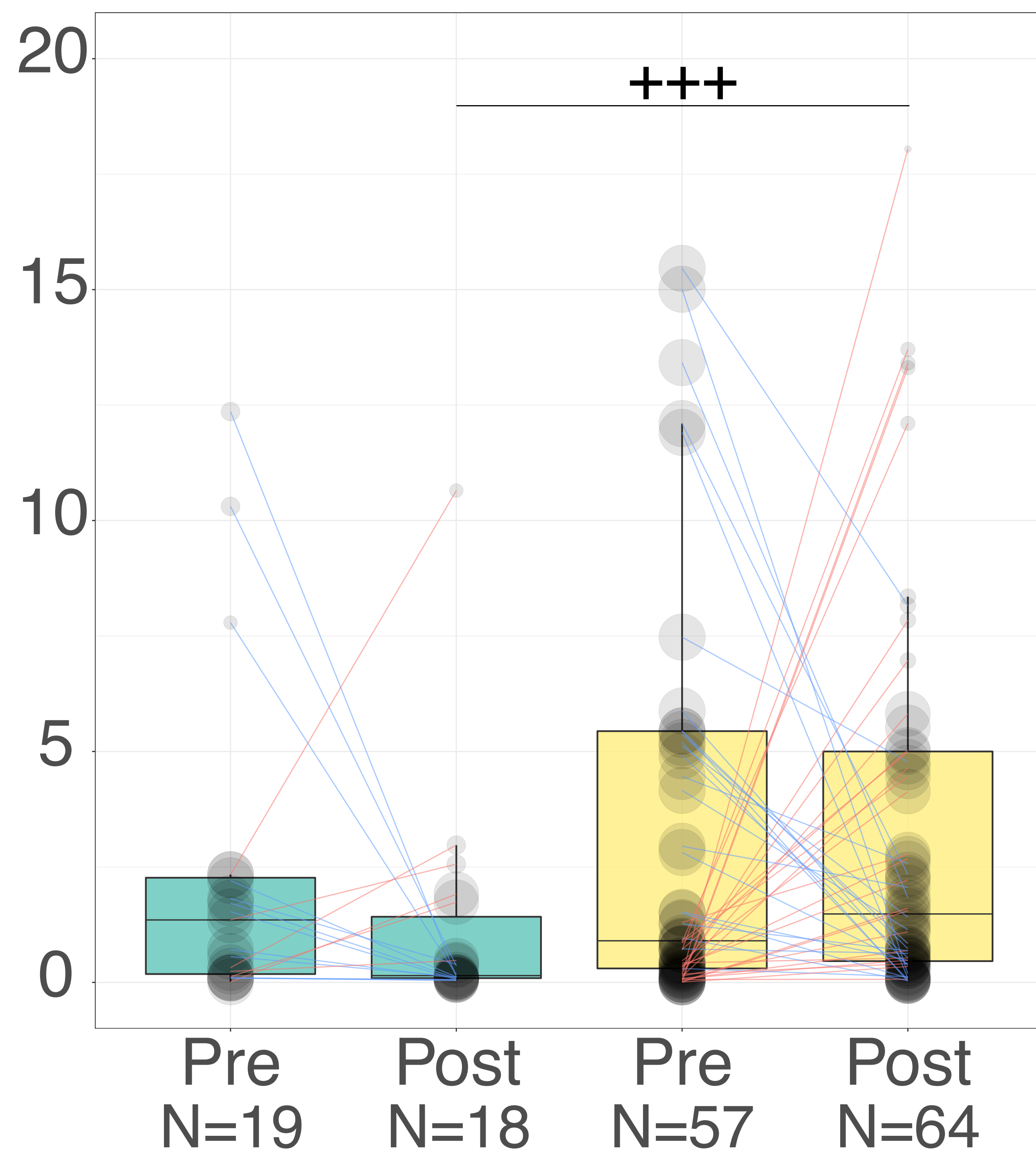

Supplement: FIG S3 [file msystems.00018-22-sf003.pdf]

**a****Pre**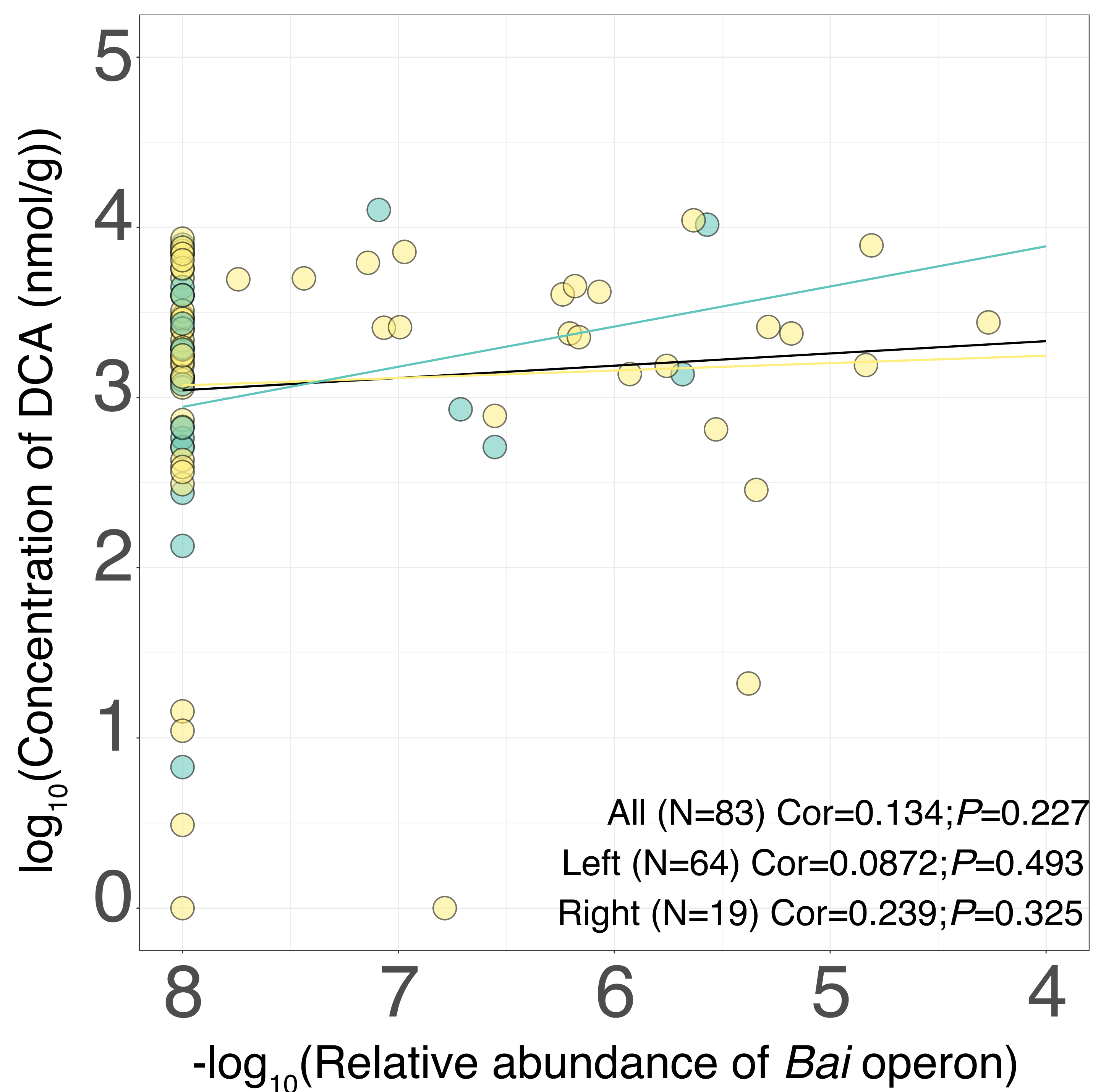**b****Post**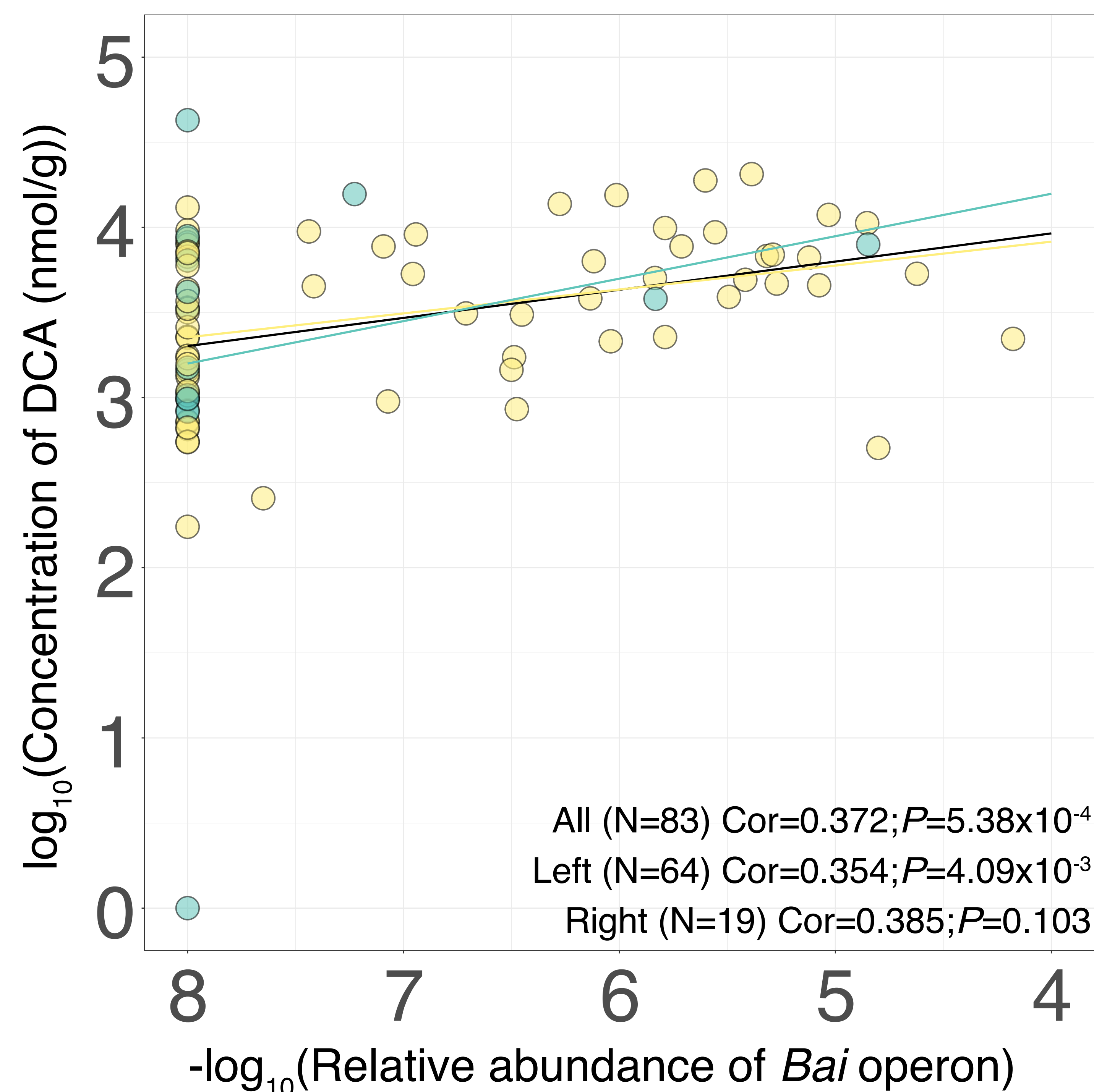**c****Pre**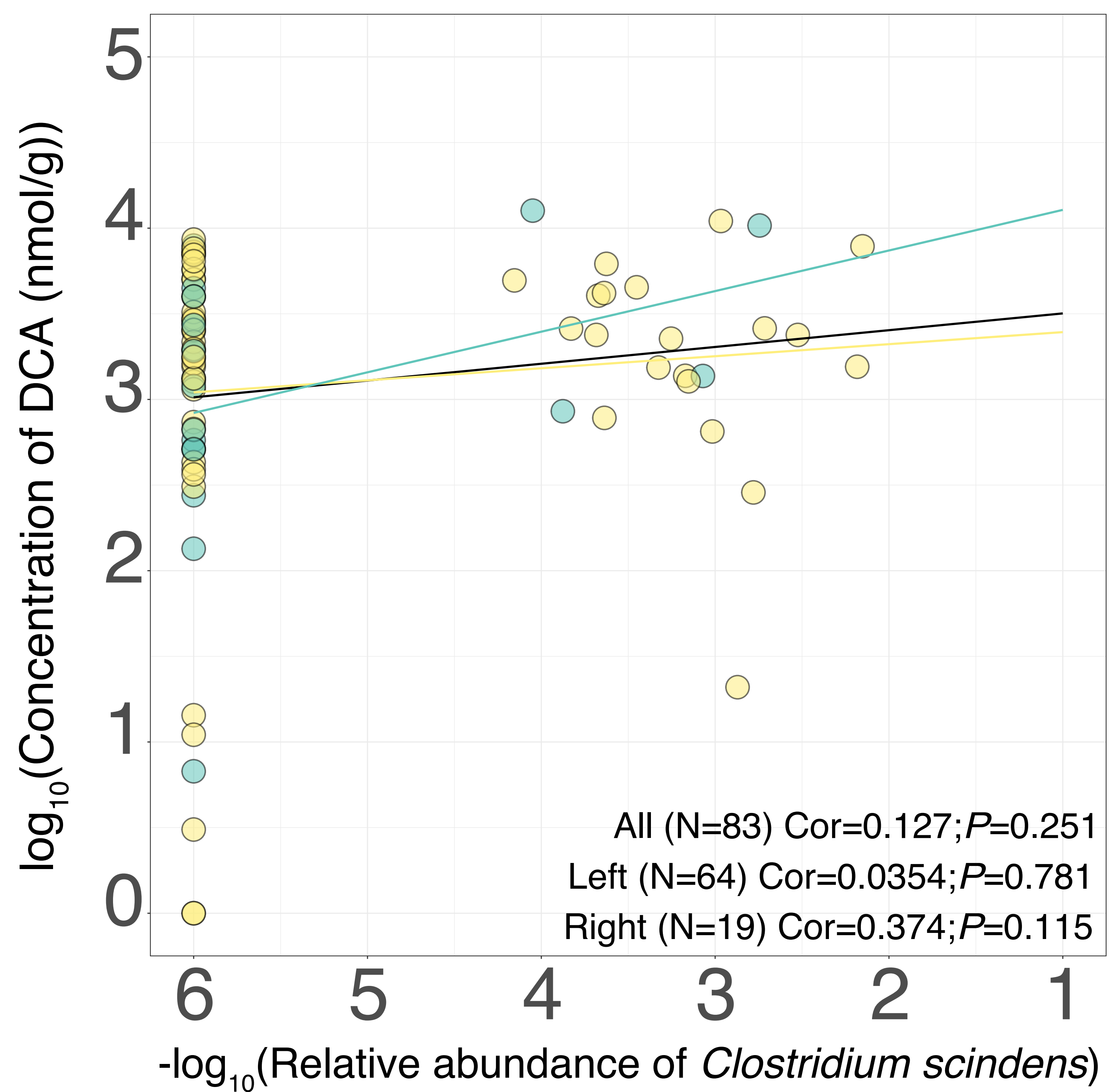**d****Post**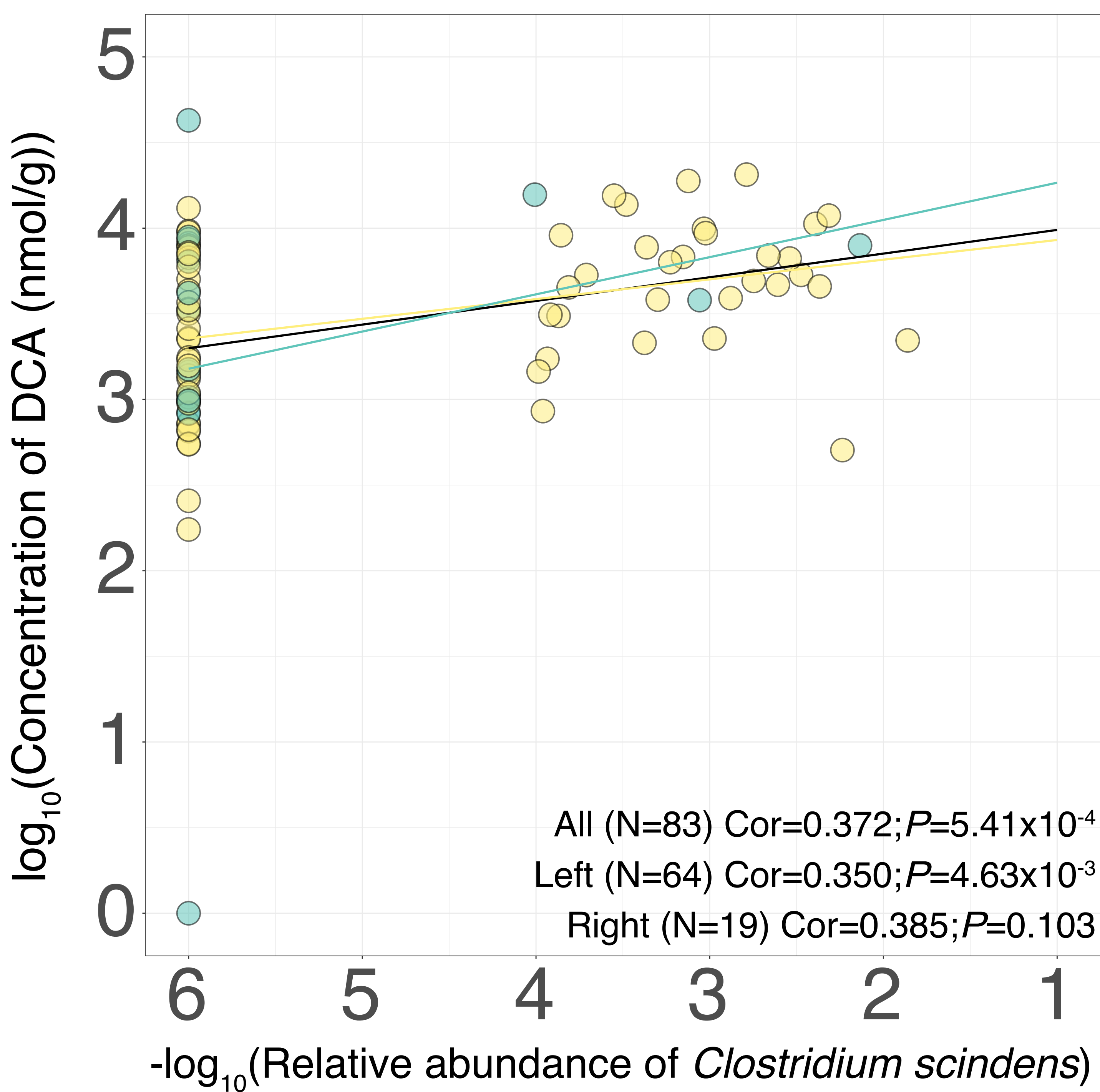

Supplement: FIG S4 [file msystems.00018-22-sf004.pdf]

**a****Species**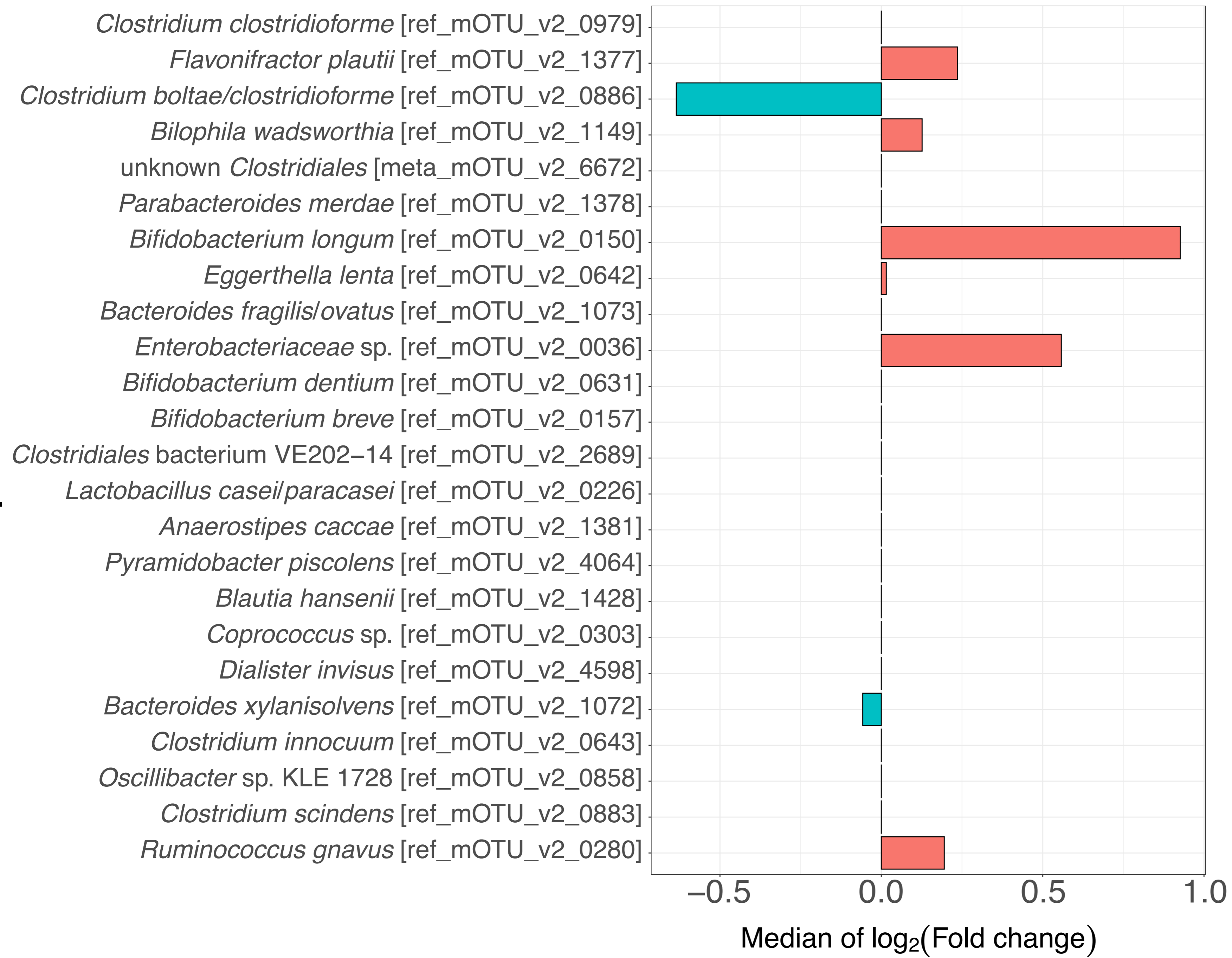**b****Metabolites**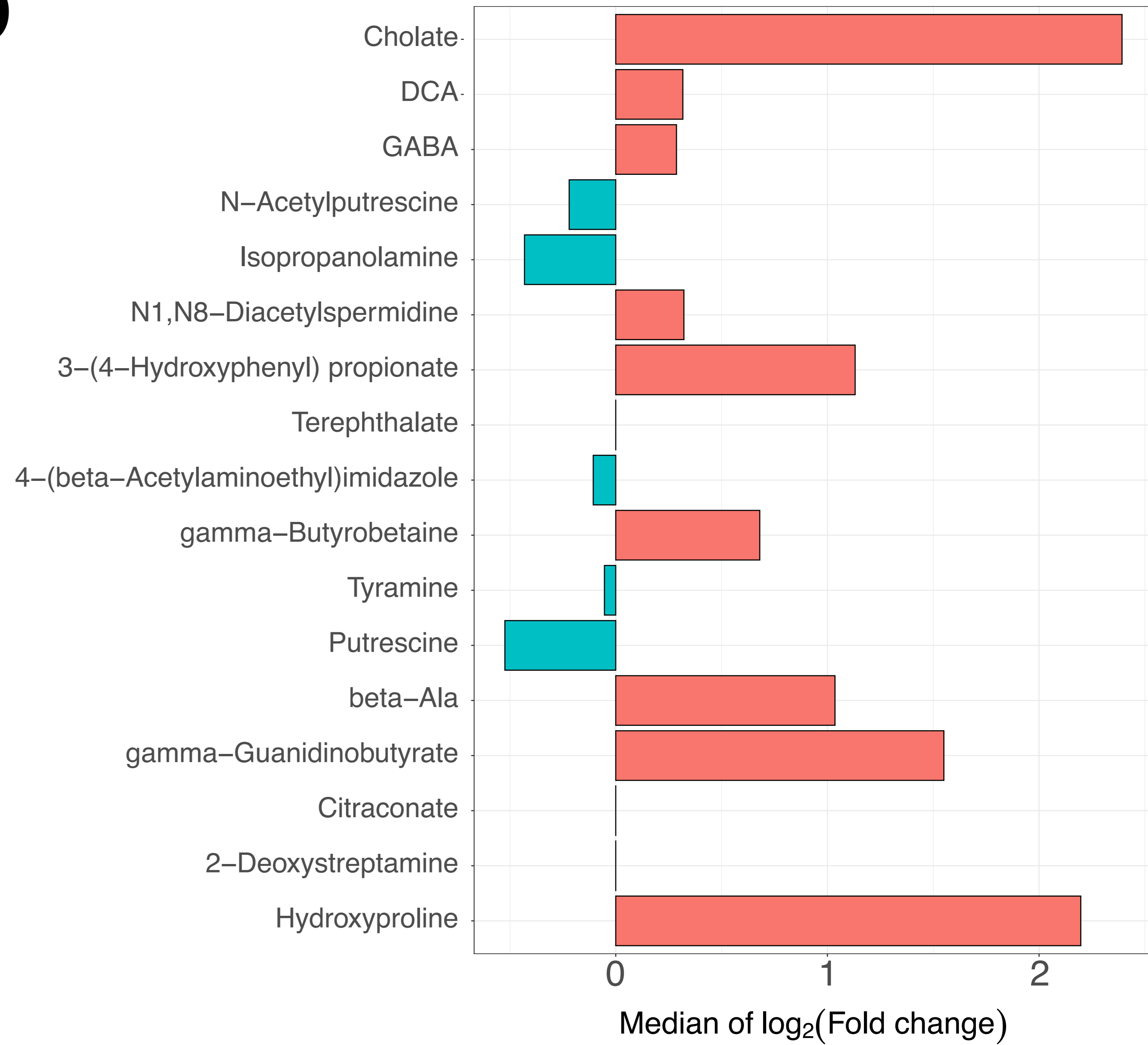

Supplement: FIG S5 [file msystems.00018-22-sf005.pdf]
