# Supplementary material for: Surgical Treatment for Colorectal Cancer Partially Restores Gut Microbiome and Metabolome Traits
Source: mSystems. 2022 Mar 21;7(2):e00018-22. doi: 10.1128/msystems.00018-22 (PMC9040882; doi:10.1128/msystems.00018-22)
Supplement: TEXT S1 [file msystems.00018-22-s0001.docx]

SUPPLEMENTARY METHODS

## DNA extraction and Sequencing

Genomic DNA was extracted from fecal samples with bead beating using a GENOME® DNA Isolation Kit (MP Biomedicals, Santa Ana, CA, USA). DNA quality was evaluated by a 4200 TapeStation (Agilent Technology, Santa Clara, CA, USA). The sequencing library was generated by the Nextera XT DNA Library Prep Kit (Illumina, San Diego, CA, USA). Library quality was evaluated by a 4200 TapeStation. Whole-genome shotgun sequencing was performed with the HiSeq 2500 platform with 150 bp paired-end read length and targeted 5 Gbps sequencing depth.

## Reads quality filtering

We subsequently obtained high-quality reads from raw reads by the following steps (**Figure S1c**):

1. Reads that included the letter ‘N’ (base pair not identified) were eliminated.
2. Reads that were mapped to the *PhiX* reference sequence using Bowtie2 version 2.2.9[(1)](https://paperpile.com/c/6ajj5K/e8D7Y) with the parameter --fast-local were eliminated.
3. The adapter sequences were removed by cutadapt version 1.9.1[(2)](https://paperpile.com/c/6ajj5K/tX6dQ) with the parameter -a CTGTCTCTTATACACATCTCCGAGCCCACGAGAC -O 33 -q 17 for the forward primer sequence and -a CTGTCTCTTATACACATCTGACGCTGCCGACGA -O 32 -q 17 for the reverse primer sequence.
4. Reads with lengths less than 50 bp were eliminated.
5. Reads with average quality scores less than 25 were eliminated.
6. Reads that were mapped to the human genome sequence reference (24 gi numbers: from 56833600 to 568336023, GRCh38) using Bowtie2 version 2.2.9 with the parameters --fast-local were eliminated.
7. Nonpaired reads were eliminated.

## Taxonomic profiling

Taxonomic profiles were generated by mOTUs2 profiler version 2.0.0[(3)](https://paperpile.com/c/6ajj5K/ZRbBw). High-quality reads were mapped against 10 universal single-copy marker gene families by the motus map_tax module in mOTUs2 with default parameters. The number of reads that were mapped to each marker gene was calculated by the motus calc_mgc module with default parameters. mOTU profiles were generated by the motus calc_motu module with -k parameters.

## Generation of metagenome-assembled genomes

Each of 610 metagenomic samples was assembled by SPAdes version 3.12.0[(4)](https://paperpile.com/c/6ajj5K/CZDQn) with the parameter --meta -t 4. Each of one hundred and six samples that failed to be processed due to computational resources (900 G of RAM and > 24 h run time using 4 threads) was assembled by MEGAHIT version 1.1.3[(5)](https://paperpile.com/c/6ajj5K/YBOgP) with the parameter --min-contig-len 1500. Short-length contigs (<1500 bp) were filtered out by seqkit version 0.10.1[(6)](https://paperpile.com/c/6ajj5K/W6d5G) with parameter seq -m 1500.

To obtain MAGs, we used metaWRAP version 1.2.1[(7)](https://paperpile.com/c/6ajj5K/SWcTo). To improve the genome quality of MAGs, we applied the metaWRAP approach, which consolidated a number of different binning results for choosing the best quality MAGs. First, we performed binning using metaWRAP with the parameters -l 1500, --metabat2, --maxbin2, and --concoct. High-quality reads were mapped against contigs using the Burrows-Wheeler Aligner MEM algorithm version 0.7.17-r1188[(8)](https://paperpile.com/c/6ajj5K/vaWTL) with default parameters. We used three different binning tools, namely, MetaBAT2 version 2.12.1[(9)](https://paperpile.com/c/6ajj5K/7Kwva), CONCOCT version 1.0.0[(10)](https://paperpile.com/c/6ajj5K/tPb8W), and MaxBin2 version 2.2.6[(11)](https://paperpile.com/c/6ajj5K/GlN4X) , to obtain MAGs. Next, refinement was carried out by the bin_refinement module from metaWRAP with the parameter -c 50 -x 10 --quick. Hybridized MAGs from all possible combinations of sets were generated by binning _refiner[(12)](https://paperpile.com/c/6ajj5K/RhWjG). Seven MAG sets (three original and four hybridized) were evaluated for genome quality (completeness rate and contamination rate) by the lineage_wf workflow from CheckM version 1.0.12[(13)](https://paperpile.com/c/6ajj5K/X7xnY) with the parameter --reduced_tree. The same MAGs were identified based on a minimum of 80% overlap in genome length. The best quality MAGs in 7 MAGs sets were determined based on the quality score (completeness rate - 5 × contamination rate). Very-low-quality MAGs (completeness rate < 50 and contamination rate > 10) were disregarded. Finally, we obtained 12399 high-quality MAGs and 10148 low-quality MAGs [cutoffs: completeness rate > 90, contamination rate < 5].

## Genome clustering

To reduce computational resources, we clustered 22547 MAGs at an estimated species level based on the average nucleotide identity (ANI) >= 95% using a dereplicate module from dRep version 2.2.3[(14)](https://paperpile.com/c/6ajj5K/ilRaW) with parameter --S_ani 0.95. First, all MAGs were compared based on MinHash to obtain Mash distance by Mash version 2.2[(15)](https://paperpile.com/c/6ajj5K/H8gIC). Each MAG set with at least 90% Mash distance was then compared in the MAG sets by Nucmer from MUMmer version 3.1[(16)](https://paperpile.com/c/6ajj5K/UFRNh) to obtain ANI. Finally, all MAGs were clustered by at least 95% of the ANI score. The species-level representative genome in each iteration was chosen as the maximized genome using the following formula:

Score = completeness rate - 5 × contamination rate + 0.5 × log (N50)

Because of the lack of computational resources for clustering the entire genome set, we randomly picked 5000 genomes and then clustered them independently. The representative genomes among 5000 genome sets were merged and then clustered to obtain the representative genomes. We obtained 1302 representative MAGs. Among them, eight hundred and seventy-nine representative MAGs were high-quality MAGs.

## Taxonomic assignment for MAGs

The taxonomic assignment for each representative MAG was performed by the classify_wf function from the GTDB-Tk module version 1.3.0[(17)](https://paperpile.com/c/6ajj5K/2JgZ5). Version 95 of GTDB-Tk was used as a reference DB. We also converted species names derived from GTDB to NCBI taxonomy by ’‘gtdb_to_ncbi_majority_vote.py’ based on the GTDB-Tk repository (<https://github.com/Ecogenomics/GTDBTk/>).

## Gene prediction and gene catalog

Gene prediction for high-quality MAGs was performed by Prokka (Prodigal) version 1.14.0[(18)](https://paperpile.com/c/6ajj5K/2Eeft) with default parameters. To obtain the catalog of MAG-driven genes, 50.3 M predicted genes were merged from all samples and clustered at 95% sequence identity and 90% length coverage using CD-HIT EST version 4.7[(19)](https://paperpile.com/c/6ajj5K/IK0yi) with the parameters -c 0.95 -G 0 -aS 0.9 -g 1 -r 1 -d -M 0. As a result, we obtained 3.41 M nonredundant genes.

To evaluate the overlap between the reference gene catalog of the human gut microbiome and our gene catalog, we merged 9.88 M reference genes[(20)](https://paperpile.com/c/6ajj5K/QKw5F) and 4.11 M nonredundant genes and clustered them using CD-HIT EST with the parameters -c 0.95 -G 0 -aS 0.9 -g 1 -r 1 -d -M 0. We confirmed that 2.84 M genes were shared by both gene sets. In addition, 6.83 M and 1.34 M genes were unique to the reference genes and our own dataset, respectively.

## Functional annotation of the gene catalog

To obtain the KO of each gene, the amino acid sequences were translated from the representative genes and annotated by the prokaryotic KEGG database (as of 2017)[(21)](https://paperpile.com/c/6ajj5K/lBvMl) using DIAMOND version 0.9.14.115[(22)](https://paperpile.com/c/6ajj5K/3krel) with parameter blastp --sensitive [cutoffs: identity > 40, bit score > 70, coverage > 80]. As a result, 2.72 M genes and 1.46 M KOs were annotated based on the KEGG database.

## Functional profiling

Paired-end high-quality reads were paired-end mapped against a gene catalog using the Burrows-Wheeler Aligner MEM algorithm with default parameters. Alignments were then filtered out to retain only those with sequence identity higher than 95% and a length longer than 50 bp. As a result, an average of 72.5% reads per sample were mapped against the gene catalog. To obtain each gene abundance, we counted the number of mapped reads against each gene and normalized the value to each gene length. We then calculated the relative abundance of each gene by dividing the gene abundances by the total abundances of all genes.

KO abundance was then computed as the sum of each annotated gene abundance corresponding to each KO. As more than two KEGG GENE match up to the one gene, each KO abundance was computed as the average of the gene's abundance. Finally, we obtained 6081 KOs in 716 samples.

## Quantification of *bai* operon from gene profile

We investigated the relative abundance of DCA (deoxycholate)-producing genes (*bai* operon) from the gene profile. First, we collected complete sequences of eight *bai* genes (*bai B*, *CD*, *E*, *A*, *F*, *G*, *H*, and *I*) from UniProtKB (as of 2020) to construct hidden Markov models (HMMs). Each gene was aligned by mafft version 7.427[(23)](https://paperpile.com/c/6ajj5K/0iJUR) and then trimmed by trimAI version 1.2rev59[(24)](https://paperpile.com/c/6ajj5K/utV2j) with the parameters -gt 0.9 -cons 60. Each trimmed sequence was used to construct an HMM by HMMER software version 3.2[(25)](https://paperpile.com/c/6ajj5K/IyRms).

Second, we searched for DCA-producing species. A previous study reported that six genes (*bai* *B*, *CD*, *E*, *F*, *A*, and *H*) in the *bai* operon are essential for the biotransformation of cholate to DCA[(26)](https://paperpile.com/c/6ajj5K/XPVB7). Therefore, we defined DCA-producing species as those that have these six genes. We screened each gene in the MAGs by an HMM with the parameter -E 1×10^-10^ and performed a homology search against each sequence of *bai* genes from UniProtKB by BLASTP from BLAST+ version 2.2.30+[(27)](https://paperpile.com/c/6ajj5K/xywTF) [cutoff: identity > 70]. Finally, we checked whether each MAG contained these six genes, and the gene arrangement relationship was accurate within the *bai* operon.

Finally, we annotated genes that corresponded with each gene from DCA-producing species in the gene profile. Quantification of each *bai* gene was performed by using the sum of each gene derived from seven MAGs in the MAG-based gene catalog, and then, the sum of the relative abundance of seven *bai* genes was calculated as the relative abundance of the *bai* operon.

## Quantification of fecal metabolites

Quantification of fecal metabolites was performed as previously described[(28–30)](https://paperpile.com/c/6ajj5K/lMXcQ+GpMML+ZxCct). Fecal metabolites were extracted from 10 mg of freshly thawed freeze-dried fecal samples suspended in 400 μL of 50% methanol in Milli-Q water containing internal standards (20 μM each methionine sulfone and D-camphor-10-sulfonic acid (CSA)). Three millimeters of zirconia beads (BioSpec Products, Bartlesville, OK, USA) and 100 mg of 0.1 mm zirconia/silica beads (BioSpec Products, Bartlesville, OK, USA) were added to the mixture and then subjected to 3 minutes of vigorous shaking using a Micro Smash (TOMY, Nerima, Tokyo, Japan). The suspension was centrifuged for 10 minutes at 1500 rpm. The supernatant was transferred to a 5-kDa cutoff filter column (Ultrafree MC-PLHCC 250/pk) for metabolome analysis (Human Metabolome Technologies, Tsuruoka, Yamagata, Japan). The flow-through was dried under vacuum, and the residue was dissolved in 40 μL of Milli-Q water containing reference compounds (200 μM each of 3-aminopyrrolidine and trimesate). The supernatant was filtered through a spin column to obtain extracted metabolites for CE-TOF MS analysis using an Agilent CE system (Agilent Technologies, Santa Clara, CA, USA).

The raw data were processed for metabolite quantification. The concentration (nmol) of each metabolite was computed based on the relative peak areas and the concentrations of the standard compounds and normalized using the fecal weight to obtain the amount of metabolite in each gram of sample (nmol/g).

We quantified 397 metabolites in 694 samples. Because the number of fecal samples was lacking, the fecal metabolites could not be quantified in 22 out of 716 samples.

## Publicly available metagenome data

We collected publicly available metagenome data (**Table S1**). To evaluate the changes in the fecal gut microbiome at two time points within the same healthy individuals, we downloaded the healthy cohort data used in Voigt *et al.*[(31)](https://paperpile.com/c/6ajj5K/1Vy4f) from the European Nucleotide Archive. The DNA extraction procedure (G’NOMEs kit, MP Biomedicals, Illkirch, France) and sequencing platform (Illumina HiSeq 2000/2500) in Voigt *et al*. were almost the same as those of this study. Here, we excluded samples derived from one individual ("alien") who used an antibiotic. In addition, the ‘’bugkiller-11-7-0” metagenomic sample was omitted due to failure in the ' Reads quality filtering' step. The Voigt *et al.* samples (29 paired samples from 6 individuals at two different time points) were processed by 'Reads quality filtering', 'Taxonomic profiling', and ‘Functional profiling’ steps as previously described to produce comparable taxonomic and functional profiles.

We also confirmed that the gut microbiota composition in healthy controls was relatively stable at different time points within the same individuals (**Table S1**).

## Publicly available metabolome data

We collected publicly available metabolome data (**Table S1**). To evaluate changes in the fecal metabolome profile at two time points within the same healthy individuals, we used data from Nagata *et al.*[(32)](https://paperpile.com/c/6ajj5K/1zSm3). In this study, fecal samples were collected from 8 healthy individuals before and 2 weeks after bowel cleansing. Nagata *et al*. samples (8 paired samples from 16 individuals at two different time points) were processed through 'Quantification of fecal metabolites’ as previously described to produce a comparable metabolome profile.

# REFERENCES

1. [Langmead B, Salzberg SL. 2012. Fast gapped-read alignment with Bowtie 2. Nat Methods 9:357–359.](http://paperpile.com/b/6ajj5K/e8D7Y)

2. [Martin M. 2011. Cutadapt removes adapter sequences from high-throughput sequencing reads. EMBnet.journal 17:10.](http://paperpile.com/b/6ajj5K/tX6dQ)

3. [Milanese A, Mende DR, Paoli L, Salazar G, Ruscheweyh H-J, Cuenca M, Hingamp P, Alves R, Costea PI, Coelho LP, Schmidt TSB, Almeida A, Mitchell AL, Finn RD, Huerta-Cepas J, Bork P, Zeller G, Sunagawa S. 2019. Microbial abundance, activity and population genomic profiling with mOTUs2. Nat Commun 10:1014.](http://paperpile.com/b/6ajj5K/ZRbBw)

4. [Nurk S, Meleshko D, Korobeynikov A, Pevzner PA. 2017. metaSPAdes: a new versatile metagenomic assembler. Genome Res 27:824–834.](http://paperpile.com/b/6ajj5K/CZDQn)

5. [Li D, Liu C-M, Luo R, Sadakane K, Lam T-W. 2015. MEGAHIT: an ultra-fast single-node solution for large and complex metagenomics assembly via succinct de Bruijn graph. Bioinformatics 31:1674–1676.](http://paperpile.com/b/6ajj5K/YBOgP)

6. [Shen W, Le S, Li Y, Hu F. 2016. SeqKit: A Cross-Platform and Ultrafast Toolkit for FASTA/Q File Manipulation. PLoS One 11:e0163962.](http://paperpile.com/b/6ajj5K/W6d5G)

7. [Uritskiy GV, DiRuggiero J, Taylor J. 2018. MetaWRAP—a flexible pipeline for genome-resolved metagenomic data analysis. Microbiome 6:158.](http://paperpile.com/b/6ajj5K/SWcTo)

8. [Li H, Durbin R. 2009. Fast and accurate short read alignment with Burrows-Wheeler transform. Bioinformatics 25:1754–1760.](http://paperpile.com/b/6ajj5K/vaWTL)

9. [Kang DD, Li F, Kirton E, Thomas A, Egan R, An H, Wang Z. 2019. MetaBAT 2: an adaptive binning algorithm for robust and efficient genome reconstruction from metagenome assemblies. PeerJ 7:e7359.](http://paperpile.com/b/6ajj5K/7Kwva)

10. [Alneberg J, Bjarnason BS, de Bruijn I, Schirmer M, Quick J, Ijaz UZ, Lahti L, Loman NJ, Andersson AF, Quince C. 2014. Binning metagenomic contigs by coverage and composition. Nat Methods 11:1144–1146.](http://paperpile.com/b/6ajj5K/tPb8W)

11. [Wu Y-W, Simmons BA, Singer SW. 2016. MaxBin 2.0: an automated binning algorithm to recover genomes from multiple metagenomic datasets. Bioinformatics 32:605–607.](http://paperpile.com/b/6ajj5K/GlN4X)

12. [Song W-Z, Thomas T. 2017. Binning_refiner: improving genome bins through the combination of different binning programs. Bioinformatics 33:1873–1875.](http://paperpile.com/b/6ajj5K/RhWjG)

13. [Parks DH, Imelfort M, Skennerton CT, Hugenholtz P, Tyson GW. 2015. CheckM: assessing the quality of microbial genomes recovered from isolates, single cells, and metagenomes. Genome Res 25:1043–1055.](http://paperpile.com/b/6ajj5K/X7xnY)

14. [Olm MR, Brown CT, Brooks B, Banfield JF. 2017. dRep: a tool for fast and accurate genomic comparisons that enables improved genome recovery from metagenomes through de-replication. ISME J 11:2864–2868.](http://paperpile.com/b/6ajj5K/ilRaW)

15. [Ondov BD, Treangen TJ, Melsted P, Mallonee AB, Bergman NH, Koren S, Phillippy AM. 2016. Mash: fast genome and metagenome distance estimation using MinHash. Genome Biol 17:132.](http://paperpile.com/b/6ajj5K/H8gIC)

16. [Kurtz S, Phillippy A, Delcher AL, Smoot M, Shumway M, Antonescu C, Salzberg SL. 2004. Versatile and open software for comparing large genomes. Genome Biol 5:R12.](http://paperpile.com/b/6ajj5K/UFRNh)

17. [Chaumeil P-A, Mussig AJ, Hugenholtz P, Parks DH. 2019. GTDB-Tk: a toolkit to classify genomes with the Genome Taxonomy Database. Bioinformatics](http://paperpile.com/b/6ajj5K/2JgZ5) 36:1925–1927[.](http://paperpile.com/b/6ajj5K/2JgZ5)

18. [Seemann T. 2014. Prokka: rapid prokaryotic genome annotation. Bioinformatics 30:2068–2069.](http://paperpile.com/b/6ajj5K/2Eeft)

19. [Fu L, Niu B, Zhu Z, Wu S, Li W. 2012. CD-HIT: accelerated for clustering the next-generation sequencing data. Bioinformatics 28:3150–3152.](http://paperpile.com/b/6ajj5K/IK0yi)

20. [Li J, Jia H, Cai X, Zhong H, Feng Q, Sunagawa S, Arumugam M, Kultima JR, Prifti E, Nielsen T, Juncker AS, Manichanh C, Chen B, Zhang W, Levenez F, Wang J, Xu X, Xiao L, Liang S, Zhang D, Zhang Z, Chen W, Zhao H, Al-Aama JY, Edris S, Yang H, Wang J, Hansen T, Nielsen HB, Brunak S, Kristiansen K, Guarner F, Pedersen O, Doré J, Ehrlich SD, MetaHIT Consortium, Bork P, Wang J, MetaHIT Consortium. 2014. An integrated catalog of reference genes in the human gut microbiome. Nat Biotechnol 32:834–841.](http://paperpile.com/b/6ajj5K/QKw5F)

21. [Kanehisa M and Goto S. 2000. KEGG: Kyoto Encyclopedia of Genes and Genomes. Nucleic Acids Research 28:27–30.](http://paperpile.com/b/6ajj5K/lBvMl)

22. [Buchfink B, Xie C, Huson DH. 2015. Fast and sensitive protein alignment using DIAMOND. Nat Methods 12:59–60.](http://paperpile.com/b/6ajj5K/3krel)

23. [Katoh K, Standley DM. 2013. MAFFT multiple sequence alignment software version 7: improvements in performance and usability. Mol Biol Evol 30:772–780.](http://paperpile.com/b/6ajj5K/0iJUR)

24. [Capella-Gutiérrez S, Silla-Martínez JM, Gabaldón T. 2009. trimAl: a tool for automated alignment trimming in large-scale phylogenetic analyses. Bioinformatics 25:1972–1973.](http://paperpile.com/b/6ajj5K/utV2j)

25. [Eddy SR. 2011. Accelerated Profile HMM Searches. PLoS Comput Biol 7:e1002195.](http://paperpile.com/b/6ajj5K/IyRms)

26. [Funabashi M, Grove TL, Wang M, Varma Y, McFadden ME, Brown LC, Guo C, Higginbottom S, Almo SC, Fischbach MA. 2020. A metabolic pathway for bile acid dehydroxylation by the gut microbiome. Nature 582:566–570.](http://paperpile.com/b/6ajj5K/XPVB7)

27. [Camacho C, Coulouris G, Avagyan V, Ma N, Papadopoulos J, Bealer K, Madden TL. 2009. BLAST+: architecture and applications. BMC Bioinformatics 10:421.](http://paperpile.com/b/6ajj5K/xywTF)

28. [Soga T, Baran R, Suematsu M, Ueno Y, Ikeda S, Sakurakawa T, Kakazu Y, Ishikawa T, Robert M, Nishioka T, Tomita M. 2006. Differential metabolomics reveals ophthalmic acid as an oxidative stress biomarker indicating hepatic glutathione consumption. J Biol Chem 281:16768–16776.](http://paperpile.com/b/6ajj5K/lMXcQ)

29. [Soga T, Igarashi K, Ito C, Mizobuchi K, Zimmermann H-P, Tomita M. 2009. Metabolomic profiling of anionic metabolites by capillary electrophoresis mass spectrometry. Anal Chem 81:6165–6174.](http://paperpile.com/b/6ajj5K/GpMML)

30. [Ishii C, Nakanishi Y, Murakami S, Nozu R, Ueno M, Hioki K, Aw W, Hirayama A, Soga T, Ito M, Tomita M, Fukuda S. 2018. A Metabologenomic Approach Reveals Changes in the Intestinal Environment of Mice Fed on American Diet. Int J Mol Sci 19:4079.](http://paperpile.com/b/6ajj5K/ZxCct)

31. [Voigt AY, Costea PI, Kultima JR, Li SS, Zeller G, Sunagawa S, Bork P. 2015. Temporal and technical variability of human gut metagenomes. Genome Biol 16:73.](http://paperpile.com/b/6ajj5K/1Vy4f)

32. [Nagata N, Tohya M, Fukuda S, Suda W, Nishijima S, Takeuchi F, Ohsugi M, Tsujimoto T, Nakamura T, Shimomura A, Yanagisawa N, Hisada Y, Watanabe K, Imbe K, Akiyama J, Mizokami M, Miyoshi-Akiyama T, Uemura N, Hattori M. 2019. Effects of bowel preparation on the human gut microbiome and metabolome. Scientific Reports 9:4042.](http://paperpile.com/b/6ajj5K/1zSm3)
